# Patient-Reported Outcome Measures in Liver and Gastrointestinal Cancer Randomized Controlled Trials

**DOI:** 10.3390/ijerph20136293

**Published:** 2023-07-04

**Authors:** Carolin Winkelmann, Anna Mezentseva, Bodo Vogt, Thomas Neumann

**Affiliations:** 1 Chair in Empirical Economics, Otto-von-Guericke-University Magdeburg, Universitätsplatz 2, 39106 Magdeburg, Germany; 2 Research Campus STIMULATE, Otto-von-Guericke-University Magdeburg, Otto-Hahn-Straße 2, 39106 Magdeburg, Germany; 3 Chair in Health Economics, Institute of Social Medicine and Health Systems Research, Otto-von-Guericke-University Magdeburg, Leipziger Str. 44, 39120 Magdeburg, Germany; 4 Chair in Health Services Research, Department of Digital Health Sciences and Biomedicine, School of Life Sciences, University of Siegen, Am Eichenhang 50, 57076 Siegen, Germany; 5 University Department of Neurology, Otto-von-Guericke-University Magdeburg, Leipziger Str. 44, 39120 Magdeburg, Germany

**Keywords:** patient-reported outcome measures (PROMs), patient-reported outcome (PRO), quality of life (QoL), health-related quality of life (HRQoL), liver cancer, gastrointestinal cancer, RCT, randomized controlled trial

## Abstract

Objective: For many years, outcomes such as mortality and morbidity were the standard for evaluating oncological treatment effectiveness. With the introduction of patient-reported outcome measures (PROMs), the focus shifted from a mere extension of a patient’s life or release from disease to the improvement of a multilayered concept of health, decisively affecting life satisfaction. In this study, we deal with the topic of PROMs in liver and gastrointestinal randomized controlled trials. Results: The final database included 43 papers reporting results of randomized controlled trials (RCTs) for liver or gastrointestinal cancer interventions where one of the primary or secondary outcomes was a health-related quality of life measure. The most often used PROM was the European Organization for Research and Treatment of Cancer Quality of Life questionnaire (EORTC QLQ-C30) for both liver cancer and gastrointestinal cancer (in 62% of gastrointestinal cancer studies and 57% of liver cancer studies). For the gastrointestinal cancer group, the QLQ-STO22, a cancer-specific extension of the QLQ-C30, was the second most commonly used PROM. In liver cancer, the generic PROM Short Form 36 and the EORTC QLQ-HCC18, a cancer-specific extension of the QLQ-C30, were the second most commonly used PROMs. Conclusion: We found that RCTs often do not include comprehensive quality-of-life measures. When quality of life is part of an RCT, it is often only a secondary outcome. For a holistic view of the patient, a stronger integration and weighting of patient-reported outcomes in RCTs would be desirable.

## 1. Introduction

An efficient allocation of limited resources in medical care and health services requires an evaluation of the effectiveness of treatments based on specific outcomes [1]. Randomized controlled trials (RCTs) are generally recognized as the most appropriate instrument in prospective studies to measure interventions’ effectiveness [2,3]. For many years, outcomes such as mortality and morbidity were the standard for evaluating the effectiveness of oncological treatment [4]. However, with the introduction of patient-reported outcome measures (PROMs), the focus shifted from a mere extension of a patient’s life or release from disease to the improvement of a multilayered concept of health, decisively affecting life satisfaction. According to the World Health Organization’s definition, health is “a state of complete physical, mental and social well-being” [5].

Several PROMs have been developed to measure patient-reported outcomes (PROs). Among the most frequently measured constructs are health status, quality of life (QoL), health-related quality of life (HRQoL), well-being, treatment satisfaction, symptoms, and functioning. The multidimensional construct of HRQoL is a logical extension of the WHO’s definition of health. It includes aspects of psychological, social, and physical functioning, and reflects the patients’ subjective assessments of their well-being [6]. According to Montazeri [7] and Coates et al. [8], HRQoL is a frequent endpoint in cancer clinical trials, either primary or secondary, and influences the development of appropriate treatments.

In a 2016 literature review, Weingärtner et al. [9] found “that PRO are inadequately assessed and reported in general clinical cancer research” (p. 825). To come to this conclusion, Weingärtner et al. [9] reviewed studies published in the years 2010–2013, and examined how and to what extent PROs are considered in advanced cancer therapy RCTs. In terms of PROMs, Weingärtner et al. [9] found that the most used PROMs in those years were Functional Assessment of Cancer Therapy (FACT) questionnaires, the European Organization for Research and Treatment of Cancer Quality of Life questionnaire (EORTC QLQ-C30), and the EuroQoL 5D.

Following the study by Weingärtner et al. [9], the main objective of the literature review in this paper was to fill the knowledge gap of the last decade. In addition, we aimed to determine whether there have been changes in the popularity of patient-reported outcome measures (PROMs).

After conducting a literature search on all types of cancer, we focused on liver and gastrointestinal cancer. In Germany, the incidence rate of hepatic cellular carcinoma (HCC) was approximately 9500 cases in 2022 [10] and the prognosis remains poor [11], with mortality rates of 8.0% for men and 3.0% for women [10]. A study conducted by the Robert Koch Institute revealed a general increase in the incidence rate of hepatic cancer [12]. With the number of deaths surpassing 800,000 annually and continuing to rise, HCC is on the verge of becoming one of the top three causes of cancer-related death [13]. Furthermore, it is important to note that not only primary malignancies are significant since approximately two-thirds of patients with liver metastasis from colorectal carcinoma die due to liver-related complications [14].

Against this background, we raised the following questions: (a) How common are QoL or HRQoL measurements in RCTs, and to what degree are liver and gastrointestinal cancer represented in the population of RCTs measuring QoL? (b) Which PROMs are used to measure QoL or HRQoL in RCTs? Furthermore, we investigated the functional focus of PROMs according to the WHO’s definition of health. In this regard, we ask a third question: (c) Which functional aspects of HRQoL are addressed through the various items of different PROMs?

## 2. Methods

We conducted a literature review on the use of PROMs in RCTs to assess HRQoL as an outcome of interventions for patients with cancer. Starting with a literature search for general cancer RCTs, we focused our research questions and detailed evaluations on the specifics of liver and gastrointestinal cancer. We focused on cancers of the liver and gastrointestinal tract because the liver is a common site for the spread of cancers of various tumor types, including those originating in the gastrointestinal tract [15,16]. In contrast, hepatocellular carcinoma, the main form of primary liver cancer, arises mainly in the liver itself [10]. In this area of cancer research, there is a strong focus on the further development of minimally invasive therapies, suggesting that RCT studies are particularly important in this area. This study did not need institutional review board approval. We used the PRISMA checklist [17] as a guideline for structuring our review.

### 2.1. Study Eligibility

This study included English language RCTs that included clinical outcomes and patient-reported HRQoL data, considered as primary or secondary endpoints. We considered journal publications (excluding systematic reviews and meta-analyses) published between January 2014 and December 2022, with adult patients with cancer only, with a diagnosis of cancer disease, not accounting for the stage of cancer. Thus, any studies on RCTs that compared types of cancer treatment and symptom management were eligible. Furthermore, it was important that the articles provided information on which PROM (i.e., which survey or questionnaire) was used to measure QoL or HRQoL. Moreover, we selected only those studies in which patients (or relatives/proxies) self-reported the PROs. Because several studies have shown that physicians’ and patients’ perceptions of the disease and treatment do not necessarily match [18,19], we excluded studies that evaluated HRQoL solely from the perspective of physicians—for example, with the Karnofsky performance status index. In addition, we excluded studies in which the PROs were not health-related and, thus, were not used in a medical setting, or did not provide complete information on the instruments or results of the PROM. Lastly, we included a PREFS rating, developed by Joy et al. [20]. PREFS is a checklist to assess the quality of studies based on five specific parameters (purpose, respondents, explanation, findings, and significance). Only those with a PREFS score higher than or equal to 3 [20] met the inclusion criteria.

### 2.2. Study Identification and Search Strategy

Considering the technical supplement suggestions to the Cochrane Handbook for Systematic Reviews of Interventions [21], we conducted a search in PubMed, CENTRAL, Science Direct, Scopus, ProQuest, and EBSCOhost. With reference to the PICOs framework [22], we designed our search strategy with a focus on three categories, (a) disease (cancer), (b) outcome (QoL and HRQoL), and (c) study design (RCT), aiming to identify studies that measured HRQoL related to cancer interventions (see search strategy in Supplementary Materials Tables S1 and S2). The literature review was performed in two phases. In the first phase, we searched the literature published in 2010–2020, and in the second phase, we added the years 2021–2022.

### 2.3. Study Selection

To detect and remove duplicates, we uploaded the initially identified set of articles into Citavi 6 (Swiss Academic Software GmbH, Wädenswil, Switzerland). Then, two reviewers (A.M.) and (C.W.) assessed the remaining initial article set by title and abstract regarding their eligibility for inclusion. The first period was evaluated by A. M. and the second period was evaluated by C.W. In case of ambiguity, a third reviewer (T.N.) assessed the articles, and conflicting views were resolved by discussion. For the resulting set of potentially relevant papers, we searched for the full-text versions of the publications and imported them into Citavi. Two of the authors again performed the subsequent full-text analysis. A.M. reviewed the first articles in the first period and C.W. in the second review period. We created a standardized data extraction sheet to collect information for further analyses. In addition to primary data (e.g., title, author, and publication type), we obtained general data (e.g., type of cancer, disease stage, and objective), specific data (e.g., treatment technique and number of participants), and PRO data (e.g., primary or secondary QoL outcome, PROM description, and number of items).

Moreover, we assessed the eligible papers’ quality using the PREFS quality measure [20]. We calculated an individual PREFS score for each study. Therefore, we adapted the PREFS quality assessment to our context of PROMs. After this adaptation, a paper could receive a maximum of 5 points—1 point for each of the assessed categories (i.e., purpose, respondents, explanation, findings, and significance). In line with Joy et al. [20], we assessed studies with a PREFS score equal to or higher than 3.0 as of sufficient quality for further consideration.

### 2.4. Synthesis Methods

In the course of an exploratory data analysis, we applied appropriate filters to the previously created data extraction sheet and assessed the frequency of certain data. We assessed the number of papers per cancer type, the frequency of PROMs, and the quantity of PROMs per study.

For an investigation of the popularity of items in the dataset, we standardized and grouped item names with the same or a similar meaning (e.g., fatigue and lack of energy). Based on this, we analyzed whether the items could be categorized into different HRQoL-related functional groups. Subsequently, we assessed the number of items per PROM type functional focus across 17 PROM types, as well as the frequency distribution of items per study.

## 3. Results

### 3.1. Study Selection 2010–2020

**Figure 1:**
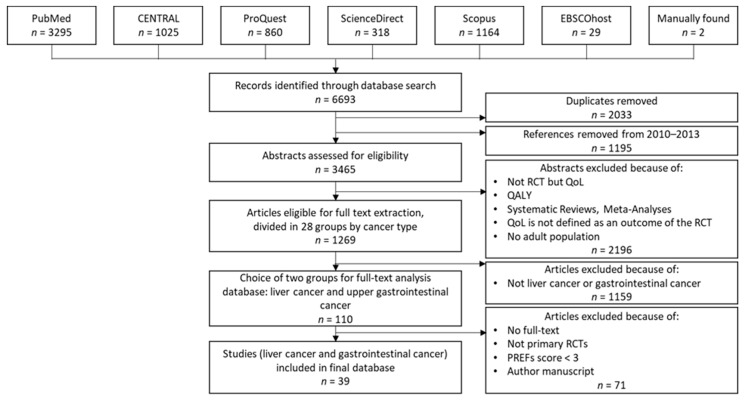
Dataset creation process in the first phase.

In the first phase of our review, we created a dataset of 39 studies for further analysis. Figure 1 shows the dataset creation process, based on the nine steps described above.

In the first step, we searched the previously mentioned databases and retrieved 6693 studies, divided among the databases as shown in Figure 1. After removing duplicates and references from 2010–2013, which Weingärtner et al. [9] had already reviewed, 3465 articles remained for further analysis. After assessing the 3465 articles by title and abstract for inclusion/exclusion criteria, a set of 1269 papers remained.

In the next step, we grouped the remaining papers according to 28 cancer types. For the following full-text analysis, we focused on 110 papers from the two groups of our field of research, liver and gastrointestinal cancer.

During full-text analysis, we eliminated 71 papers. For two papers, no full text was available. Twenty-five papers were eliminated because they did not meet the inclusion criteria based on study design details that were not apparent in the abstract analysis. We excluded one paper because it was an author’s manuscript, meaning a paper accepted for publication but not published yet. Forty-three papers did not meet the PREFS threshold of 3 points [20]. We considered the remaining 39 papers for our final evaluations.

**Figure 2:**
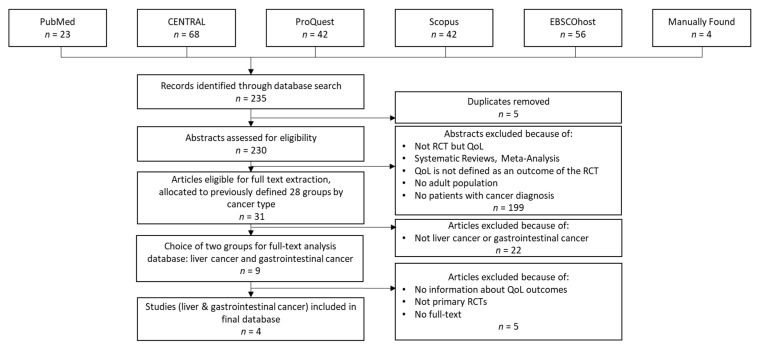
Dataset creation process in the second phase.

For internal reasons, we replicated the study selection process in early 2023 to consider papers published in 2021 and 2022 (i.e., the second phase of our review). Figure 2 summarizes the dataset creation process in the second phase of our review.

As Figure 2 shows, the procedure was similar to that performed in the first phase. However, due to changes in the compilation of the databases or in the journals indexed in them, some differences occurred. The changes in ScienceDirect should be mentioned here. The extension of this database led to a significant increase in the hits we received (approximately 1050 hits per year compared to 30 hits per year in the first search). In addition, due to the current lack of an agreement between Elsevier and German universities preventing access to these publications, we decided to exclude this database for the second phase of our review.

During the second-phase database search, we found 235 articles. After removing duplicates and reviewing the abstracts and titles, we selected 31 articles and classified them into the previously defined cancer-type groups. As a result, we additionally considered four articles within this review.

### 3.2. Results of Syntheses

**Figure 3:**
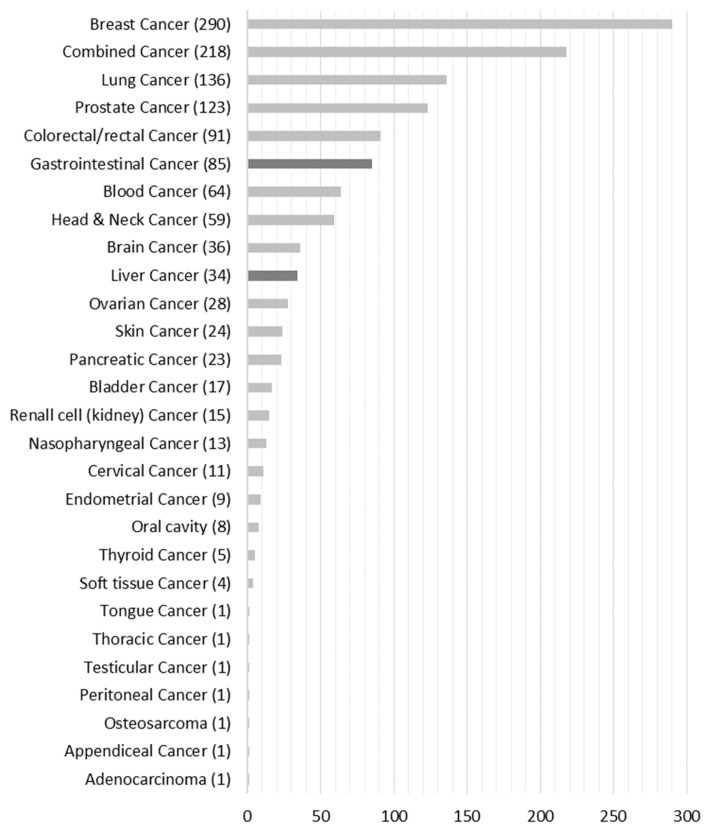
Number of papers grouped according to cancer type.

Figure 3 shows that 1300 publications qualified for full-text analysis that we assigned to the respective 28 cancer-type groups. For full-text analysis, we focused on the liver (*n* = 34) and gastrointestinal (*n* = 85) cancer groups, which accounted for 9.2% of the total population.

### 3.3. Distribution of Publications by Cancer Type

The final database included 43 papers reporting results of RCTs for liver or gastrointestinal cancer interventions where one of the primary or secondary outcomes is a HRQoL measure. The most frequent cancer type was gastrointestinal cancer (29 out of 43). One paper concentrated on two types of cancer, gastric and colorectal, with a clear differentiation of the results [23]. Table 1 additionally specifies the two cancer groups and subdivides them into further subgroups.

### 3.4. Frequency of PROM Types Used in Liver and Gastrointestinal Cancer Studies

**Figure 4:**
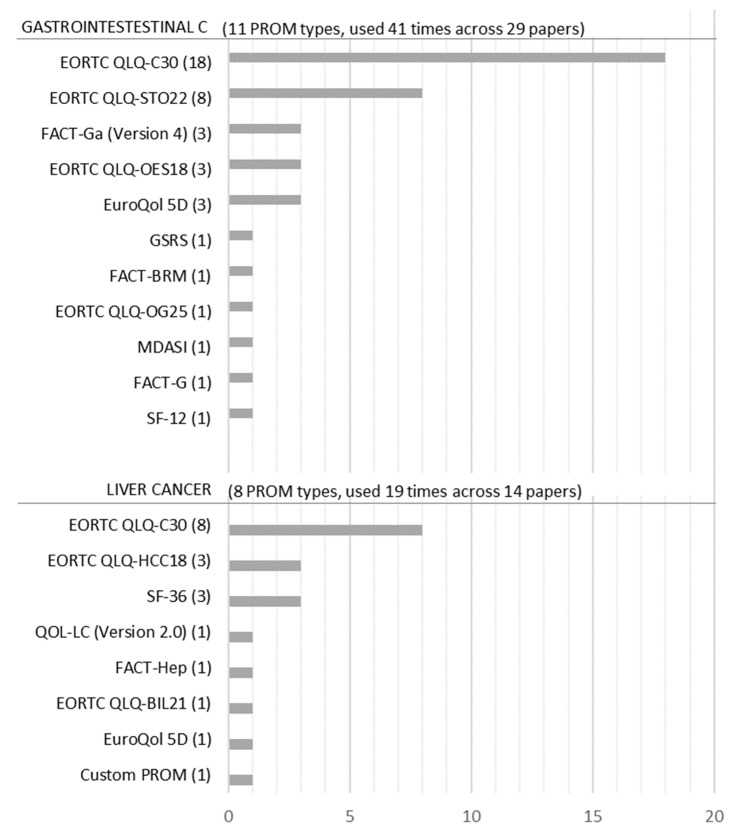
Frequency of PROMs for liver and gastrointestinal cancer.

Figure 4 shows that 19 PROM types were used 60 times across 43 studies. However, because two PROMs (EORTC QLQ-C30 and EuroQoL 5D) were used for both gastrointestinal and liver cancer studies, we only obtained 17 PROM types. Within the 17 PROM types, EuroQoL 5D, SF-36, and SF-12 are considered generic PROMs [24], even though in this dataset SF-36 was only used for liver cancer. PROMs such as EORTC QLQ-C30, FACT-G, and the MD Anderson Symptom Inventory (MDASI) are considered general cancer PROMs [25,26,27].

Figure 4 also shows that a distinct set of PROMs has been applied to the different cancer-type studies. Eleven PROM types were used for gastrointestinal cancer and eight types were used for liver cancer. The only PROMs that are applied to both cancer types were EORTC QLQ-C30 and the EuroQoL 5D (only one case for liver cancer). The most often used PROM was the EORTC QLQ-C30 for both liver cancer and gastrointestinal cancer (in 62% of gastrointestinal cancer studies and 57% of liver cancer studies). For the gastrointestinal cancer group, the QLQ-STO22, a cancer-specific extension of the QLQ-C30, was the second most commonly used PROM (28% of gastrointestinal cancer studies). In liver cancer, the generic PROM SF-36 and the EORTC QLQ-HCC18, a cancer-specific extension of the QLQ-C30, were the second most commonly used PROMs (both 21% of liver cancer studies).

### 3.5. Number of PROMs per Study

Table 2 shows that researchers used more than one PROM per study in 37% of the selected studies. Among the papers that used only one PROM, the EORTC QLQ-C30 was the most popular questionnaire. In publications using two or more PROMs, the clear tendency was to use a combination of the EORTC QLQ-C30, which measures overall QoL for cancer in general, and cancer-specific supplemental questionnaires such as the EORTC QLQ-HCC18 (for patients with hepatocellular carcinoma) and the EORTC QLQ-STO22 (gastric cancer module). The probable reason for the use of a combination of PROMs was to obtain more precise results for an RCT’s respective cancer focus.

### 3.6. Set of Items

Each PROM uses a set of items to measure QoL. To analyze the popularity of items and their importance for the assessment of QoL, we investigated the frequency of the items within the set of 43 selected studies. In total, 391 items across the 17 PROM types were evaluated 1536 times. The data showed that PROMs often use different terminology for comparable items (e.g., *fatigue* and *lack of energy*). For further analysis, we summarized the item names according to their underlying meaning. Following the WHO’s definition of health [5], we considered three functional groups: physiological functioning, mental functioning, and social functioning. An additional group labeled *general health* was used to categorize questions focusing on a subjective construct of health, such as “How would you rate your overall health during the past week on a scale of 1 to 7?” [28].

### 3.7. Frequency of Functional Groups

In Table 3, we examined the number of items across the 17 PROMs and their respective functional focus. Each PROM contained an average of 21 items, ranging from 4 to 46 items. The data showed that the physiological, mental, and social functioning groups are represented in most PROM types, but we saw an overall strong focus on physiological aspects (54% of all attributes across the PROM types). Nevertheless, differences existed between the PROM types. For example, the gastrointestinal symptom rating scale (GSRS) focuses exclusively on physiological aspects, whereas the FACT-G gives equal weight to all three functional groups.

**Figure 5:**
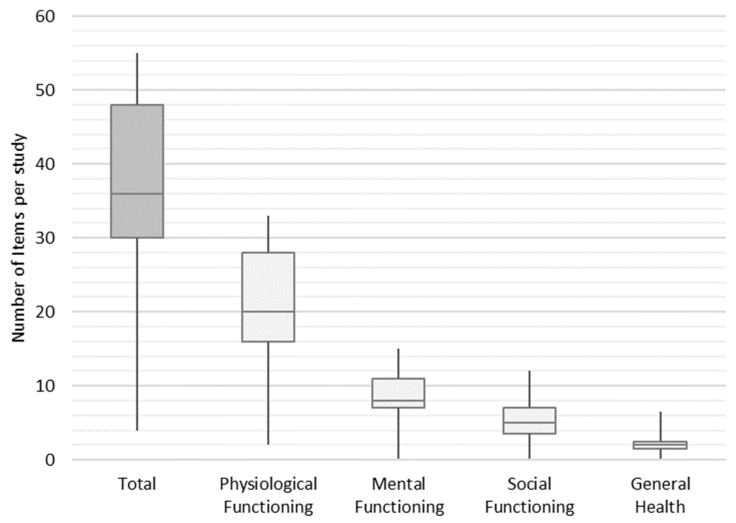
Distribution frequency of items per study.

In the next step, we weighted the items to find out how frequently they are used in an average study across the final set of studies. Each study contained an average of 36 items, of which 20 items, 8 items, 5 items, and 2 items were assigned to the functional groups of physiological functioning, mental functioning, social functioning, and general health, respectively. The minimum and maximum number of items per study ranged from 4 to 55 (shown in Figure 5).

## 4. Discussion

### 4.1. Summary of Evidence

The present paper examined the characteristics of PROMs in liver and gastrointestinal cancer RCTs. The literature showed that the terms *QoL* and *HRQoL* are interchangeable [29,30]. Our review revealed the same impression, because in almost all contributions, both terms were used.

During our abstract and title screening, we eliminated many studies because QoL was not defined as an outcome of the RCT or QoL data were not reported in the full text. In the studies we considered eligible for our review, QoL was a secondary outcome in 29 out of 43 studies. Only 10 out of 43 papers defined QoL as a primary outcome of the study (5 for liver cancer and 5 for gastrointestinal cancer). The remaining four studies defined QoL as both a primary and secondary outcome. These findings are in line with research by Brundage et al. [31], who noted that QoL is not in the primary scope of RCTs, and when QoL is included in an RCT, it is usually a secondary outcome (75% of studies) and less often a primary outcome (25% of studies) [31]. Furthermore, we confirmed Weingärtner et al.’s [9] finding that PROMs in general were also underrepresented in clinical cancer research for our review period from 2014 to 2022.

When RCTs are intended to measure QoL, attributes are not arbitrarily selected or created for individual studies, but standardized PROMs are commonly used. We found a variety of PROMs to measure QoL or HRQoL, differing in their focus from broad to narrow. General PROMs (e.g., EuroQoL 5D, SF-36, and SF-12) aim to assess the health of the population; however, these PROMS do not address a specific type of disease. More focused PROMs (e.g., EORTC QLQ-C30, FACT-G, and MDASI) specifically assess the QoL of patients with cancer. PROMs that are even more specific are explicitly designed to measure HRQoL of a specific cancer type (e.g., EORTC QLQ-HCC18, EORTC QLQ-STO22, and FACT-Ga.). This shows that a combination of PROMs may be used to obtain more precise results for an RCT’s respective cancer focus.

Looking at the popularity of PROMs, we found that the EORTC QLQ-C30 is the most often used PROM across both cancer types of liver and gastrointestinal cancer. In contrast, Weingärtner et al. [9] found the FACT questionnaire and the EuroQoL 5D to be of equal popularity to the EORTC QLQ-C30 until 2013, while we found the FACT and EuroQoL 5D to account for only 10% or less of the PROMs used from 2014 to 2022. One reason could be that the EORTC QLQ-C30 has gained more acceptance over time due to its modularity and the ability to adapt it to different cancer types, as well as its availability in multiple languages. As a general cancer PROM, the EORTC QLQ-C30 is often used in combination with cancer-specific PROMs (in 16 out of 25 applications). For liver cancer, the EORTC QLQ-C30 is combined with the EORTC QLQ HCC18, and for upper gastrointestinal tract cancers, it is combined with the EORTC QLQ STO22. Using general PROMs like the EORTC QLQ-C30 offers the advantage of greater comparability and allows high-level conclusions to be drawn from a broader range of RCTs and across different types of cancer. Extending the general PROMs with specific PROMs furthermore gives the opportunity to investigate more detailed, cancer-type-specific topics in the same study.

Following the WHO’s definition, health cannot only be described by physiological aspects, but is also influenced by mental and social aspects [5]. In a detailed examination of PROMs, we found that a large proportion covers physiological, mental, and social aspects to some extent, although there is a strong focus on physiological aspects. PROMs are applied in an area that primarily focuses on influencing physiological aspects by default, so a corresponding weighting is to be expected. Nevertheless, the question arises as to whether HRQoL in general, and the mental and social level in particular, are considered to a sufficient extent.

In conclusion, using PROMs allows researchers to focus on and to gain better insights into the patient-relevant aspects of therapies or medications, such as symptoms, side effects, functional improvement, and the resulting quality of life. Additionally, PROMs can capture long-term outcomes in RCTs with extended follow-up periods, which may not be apparent through short-term clinical assessments alone. Especially with respect to personalized treatments, PROMs provide a means to capture a patient’s needs and align individual treatment methods accordingly, tailoring them to the individual requirements of patients.

In contrast to the advantages, there are also disadvantages in the application of PROMs. The first major challenge in implementing PROMs begins with the selection of a suitable PROM for a specific condition or intervention. Choosing the right instrument across numerous validated PROMs requires careful consideration of the specific research question. Furthermore, a participant’s subjective perception regarding therapy or intervention always comes with certain biases and, therefore, might contradict the idea of evidence-based medicine.

Taking into consideration the advantages and disadvantages of PROMs, the question remains: Where does medicine want and need to develop in the future? Should there be general deductions based on evidence-based medicine for specific medical conditions in order to derive generalizable recommendations, or should the focus shift towards individualized patient care? In the context of evidence-based medicine, how does one deal with increasing individualization? One possible scenario would be to implement these PROMs in accordance with guidelines as a fixed component of RCTs, in order to bridge the gap towards personalized medicine in the future. Exploring how to consider this trade-off in line with evidence-based medicine will offer further research opportunities.

### 4.2. Limitations

A limitation of this study was the quality assessment of the papers using the PREFS score. Because the PREFS checklist [20] attempts to break down the qualities of a paper into only five dimensions, the question can be asked whether PREFS is comprehensive enough to assess all relevant aspects of report quality. Furthermore, the binary points were awarded according to a subjective evaluation of whether the respective criteria were sufficiently fulfilled for further evaluation in this review. Thus, a low PREFS score does not necessarily equate to low quality.

Another limitation of the study was the assignment of items to one of the four functional groups. Some items could be assigned to one or the other category, depending on interpretation. For example, sleep-related issues can be assigned to physiological as well as mental functioning. Interference with usual activities can be assigned to physiological (e.g., stairs or lifting) and social functioning (e.g., free time or work). Therefore, the categorization of the items was performed by two reviewers (C.W. and T.N.). This limitation also applies to PROMs in general, in that some items leave the respondents room for interpretation. If an evaluation of the PROs at the functional level is desired, it may be useful to adjust the wording of the items accordingly. It is important to ensure that the adjustment is made in such a way that the validity is not impaired. As an alternative to the adjustment of items, adding a functional PROM to the general PROM could be considered.

### 4.3. Future Research

In this review, we divided the articles into four functional groups. This allowed conclusions to be drawn about the focus of PROMs and studies, but not about the items themselves. In future research, the item level could be evaluated in detail.

## 5. Conclusions

We found that there is still no comprehensive inclusion of QoL measures in RCTs. When QoL is part of an RCT, it is often only a secondary outcome. In the sense of a holistic view of the patient, stronger integration and weighting of PROs in RCTs would be desirable. In summary, there have been no major changes in the utilization of PROs in the past 10 years, and PROs continue to be significantly underrepresented in RCTs.

## Figures and Tables

**Table 1:** Distribution of papers by cancer type.

Type of Cancer	Quantity of Papers
Liver cancer	14
Colorectal liver metastases	3
Hepatocellular carcinoma	6
Cholangiocarcinoma	1
Liver cancer	4
Gastrointestinal cancer	29
Esophageal cancer	3
Esophageal or gastric cancer	3
Gastric and colorectal cancer	1
Gastric cancer	17
Gastrointestinal cancer	3
Upper gastrointestinal cancer	2
Total	43

**Table 2:** Quantity of PROMs per study.

Type of Cancer	No. of Studies		
	One PROM	Two PROMs	Three PROMs
Colorectal liver metastases	3		
Liver cancer	6	5	
Gastrointestinal cancer	18	10	1
Total No. of studies	27	15	1

**Table 3:** Number of items per PROM type functional focus across 17 PROM types.

PROM Type	No. of Items	Physiological Functioning	Mental Functioning	Social Functioning	General Health
Custom PROM	4	50%	50%	0%	0%
EORTC QLQ-BIL21	12	67%	25%	0%	8%
EORTC QLQ-C30 (V.3)	30	53%	23%	17%	7%
EORTC QLQ-HCC18	18	67%	22%	11%	0%
EORTC QLQ-0ES18	13	92%	0%	8%	0%
EORTC QLQ-OG25	25	64%	28%	8%	0%
EORTC QLQ-STO22	22	77%	18%	5%	0%
EuroQol 5D	5	60%	20%	20%	0%
FACT-BRM (Version 4)	40	35%	38%	23%	5%
FACT-G	27	30%	30%	33%	7%
FACT-Ga (Version 4)	46	46%	24%	26%	4%
FACT-Hep	45	53%	22%	20%	4%
GSRS	15	100%	0%	0%	0%
MDASI	19	53%	26%	11%	11%
Qol-LC v2.0	22	41%	32%	23%	5%
SF-12	12	42%	33%	17%	8%
SF-36	36	56%	22%	6%	17%
**Weighted Average**	**391**	**54%**	**25%**	**16%**	**5%**

## Data Availability

The corresponding datasets of this study are available from the corresponding author upon reasonable request.

## Supplementary Materials

The following supporting information can be downloaded at: https://www.mdpi.com/article/10.3390/ijerph20136293/s1. Supplementary File Table S1: Search Queries and Additional Filters (2010–2020), Table S2: Search Queries and Additional Filters (2021–2022) and References Final Database.

## Abbreviations

HRQoL	Health-Related Quality of Life;
PRO	Patient Reported Outcome;
PROM	Patient Reported Outcome Measure;
QoL	Quality of Life;
RCT	Randomized Controlled Trial;
WHO	World Health Organization.
