# Supplementary material for: Patient-Reported Outcome Measures in Liver and Gastrointestinal Cancer Randomized Controlled Trials
Source: Int J Environ Res Public Health. 2023 Jul 4;20(13):6293. doi: 10.3390/ijerph20136293 (PMC10341660; doi:10.3390/ijerph20136293)
Supplement: Supplementary file 1 [file ijerph-20-06293-s001.zip › ijerph-2396156-supplementary.pdf]

Supplementary Materials

Patient-Reported Outcome Measures in Liver and Gastrointestinal Cancer Randomized Controlled Trials

Carolin Winkelmann, Anna Mezentseva, Bodo Vogt and Thomas Neumann

Table S1. Search Queries and Additional Filters (2010-2020).

**CENTRAL**	#1 ("randomized controlled trial"):ti,ab,kw<br>#2 (cancer OR tumour OR oncology OR neoplasms)<br>#3 (quality of life OR QoL OR QOL OR health related quality of life OR HRQoL OR health-related quality of life)<br>#4 MesH descriptor: [Neoplasms] explode all trees<br>#5 MeSH descriptor: [Quality of Life] explode all trees<br>#6 - #1 AND #2 AND #3<br>#7 - #1 AND #4 AND #5<br>#8 - #6 AND #7	**1,025 articles**
Additional filters	Trials<br>Years 2010-2020<br>Embase	
**EBSCOhost**	(((AB RCT OR TI RCT OR SU RCT) OR ((AB randomized OR TI randomized OR SU randomized) AND (AB controlled OR TI controlled OR SU controlled) AND (AB trial OR TI trial OR SU trial)) OR ((AB clinical OR TI clinical OR SU clinical) AND (AB trial OR TI trial OR SU trial))) AND (AB (cancer OR neoplasms OR oncology) OR TI (cancer OR neoplasms OR oncology) OR SU (cancer OR neoplasms OR oncology)) AND (((AB Health OR TI Health OR SU Health) AND (AB related OR TI related OR SU related) AND (AB life OR TI life OR SU life)) OR ((AB quality OR TI quality OR SU quality) AND (AB of OR TI of OR SU of) AND (AB life OR TI life OR SU life))OR ((AB patient OR TI patient OR SU patient) AND (AB reported OR TI reported OR SU reported) AND (AB outcome OR TI outcome OR SU outcome)))) AND (DT > 20100101 AND DT < 20201231)	**29 articles**
Additional filters	Filter for all three databases: Scholarly (Peer Reviewed) Journals, publication Jan10-Dec20	
**APA PsycArticles**	2010.2020<br>Method: clinical trial<br>Age Groups +18<br>Population: human<br>Psychological and Behavioral<br>Sciences Collection<br>DocType: article	
**APA PsycInfo**	2010.2020<br>English<br>Age Group: +18<br>Doc type: journal article<br>Pub type: Peer Reviewed Journal<br>Pub status: fully published<br>Population: human<br>Methodology: clinical trial	
**PSYNDEX**	2010.2020	

	English<br>Age Groups: +18<br>Doc type: journal article<br>Evidence Phase: clinical study phase 2: randomized controls	
**PubMed**	(“randomized controlled trial” [Publication Type] OR “randomized controlled trials as topic [MeSH Terms] OR “randomized controlled trials [All Fields] OR “randomised controlled trials” [All Fields]) AND (“neoplasm s” [All Fields] OR “neoplasms” [MeSH Terms] OR “neoplasms” [All Fields] OR “neoplasm” [All Fields]) AND (“quality of life [MeSH Terms] OR (“quality” [All Fields] OR “life” [All Fields] OR “quality of life” [All Fields] OR (“health” [All Fields] AND “related” [All Fields] AND “quality” [All Fields] AND “life” [All Fields]) OR “health related quality of life” [All Fields])	**3,295 articles**
Additional filters	Cancer<br>From 2010 – 2020<br>Humans<br>Adult: 19+ years<br>Randomized Controlled Trial	
**ProQuest**	(((AB(RCT) OR TI(RCT) OR SU(RCT) OR ((AB(randomized) OR TI(randomized) OR SU(randomized)) AND (AB(controlled) OR TI(controlled) OR SU(controlled)) AND (AB(trial) OR TI(trial) OR SU(trial))) OR ((AB(clinical) OR TI(clinical) OR SU(clinical) AND (AB(trial) OR TI(trial)OR SU(trial)))) AND (AB(cancer OR neoplasms OR oncology) OR TI(cancer OR neoplasms OR oncology) OR SU(cancer OR neoplasms OR oncology)) AND (((AB(Health) OR TI (Health) OR SU(Health)) AND (AB(related) OR TI(related) OR SU (related)) AND (AB(quality) OR TI(quality) OR SU(quality)) AND (AB(of) OR TI(of) OR SU(of)) AND (AB(life) OR TI(life) OR SU(life))) OR ((AB(patient) OR TI(patient) OR SU(patient) AND (AB(reported) OR TI(reported) OR SU(reported)) AND (AB(outcome) OR TI(outcome) OR SU(outcome))))) AND (YR(>=2010) AND YR(<=2020)	**860 articles**
Additional filters	Scholarly Journals<br>English<br>Article OR Case study<br>Clinical trial AND quality of life	
**ScienceDirect**	(“randomized controlled trial” OR “randomized controlled trials”) AND (“neoplasms” OR “cancer” OR “tumour” OR “oncology”) AND (“quality of life” OR “health related quality of life”)	**318 articles**
Additional filters	Years 2010-2020<br>Article type: research articles	
**Scopus**	TITLE-ABS-KEY(“randomized controlled trial” AND (cancer OR neoplasms OR oncology OR tumour) AND “health related quality of life”)	**1,164 articles**
Additional filters	2010-2020<br>Doc type: article<br>Publication stage: final<br>Source type: journal<br>English	

Table S2. Search Queries and Additional Filters (2021-2022).

**CENTRAL**	#1 ("randomized-controlled trial"):ti,ab,kw<br>#2 MeSH descriptor: [Quality of Life] explode all trees<br>#3 MeSH descriptor: [Liver Neoplasms] explode all trees<br>#4 #1 AND #2 AND #3<br>#5 (randomized controlled trial):ti,ab,kw AND (cancer) AND (health related quality of life)<br>#6 (randomized controlled trial):ti,ab,kw AND (cancer) AND (health related quality of life)	**68 articles**
Additional filters	Custom range 01/01/21 to 31/12/22<br>Topics: cancer	

**EBSCOhost**	"TI (rct or randomised control trial) OR AB (rct or randomised control trial) AND TI (health related quality of life or hrqol or quality of life or qol) OR AB (health related quality of life or hrqol or quality of life or qo) AND TI (cancer or neoplasms or oncology or tumor) OR AB (cancer or neoplasms or oncology or tumor) Full Text; Published Date: 20210101-20221231; Scholarly (Peer Reviewed) Journals; Age Groups: Adulthood (18 yrs & older); Document Type: Journal Article; Year of Publication: 2021-2022; Publication Status: Fully Published; Population Group: Human; Publication Year: 2021-2022; Publication Status: fully published; Publication Type: Peer Reviewed Journal; English; Population Group: Human; Language: English; Evidence Phase: clinical study, phase 2: randomized controls	**56 articles**
Additional filters	Filter for all three databases: Full text, Published Date: Jan 2021 – Dec 2022, Doc-type: journal article, Scholarly (peer reviewed) journals, Age groups: adulthood (18 yrs & older)	
APA PsycArticles	Year of publication: 2021-2022<br>Population group: human<br>Publication status: fully published	
APA PsycInfo	Publication year: 2021-2022<br>Publication status: fully published<br>Publication group: human<br>English	
PSYNDEX	Language: English<br>Evidence phase: clinical study, phase 2: randomized controls	
**PubMed**	("neoplasms"[All Fields] OR "cancer"[All Fields] OR "tumour"[All Fields] OR "oncology"[All Fields]) AND "quality of life"[All Fields] AND "RCT"[All Fields])	**23 articles**
Additional filters	2021-2022<br>Free full text<br>Article type: randomized controlled trial<br>Species: Humans<br>Language: English<br>Adult 19+ years	
**ProQues**t	noft(randomized controlled trial OR rct) AND (liver cancer OR oncology OR neoplasms OR tumour) AND (health related quality of life OR hrqol OR quality of life OR qol)	**42 articles**
**Additonal filters**	2021-01-01 – 2022-12-31<br>Scholarly journals<br>Article<br>English<br>clinical trials, AND quality of life, AND cancer, AND oncology	
**Scopus**	(TITLE-ABS-KEY (randomized AND controlled AND trial OR rct OR patient AND reported AND outcome) AND TITLE-ABS-KEY (cancer OR neoplasm OR oncology OR tumour) AND TITLE-ABS-KEY (health AND related AND quality AND of AND life OR quality AND of AND life OR qol)) AND (LIMIT-TO (PUBYEAR , 2021) OR LIMIT-TO (PUBYEAR , 2022)) AND (LIMIT-TO (EXACTKEYWORD , "Neoplasms")) AND (LIMIT-TO (DOCTYPE , "ar")) AND (LIMIT-TO (LANGUAGE , "English")) AND (LIMIT-TO (PUBSTAGE , "final")) AND (LIMIT-TO (SUBJAREA , "MEDI"))	**42 articles**

References Final Database

Bodoky, G.; Scheulen, M. E.; Rivera, F.; Jassem, J.; Carrato, A.; Moiseyenko, V.; Vynnychenko, I.; Prausová, J.; van Laethem, J.-L.; Cascinu, S.; Ajani, J. A. Clinical Benefit and Health-Related Quality of Life Assessment in Patients Treated with Cisplatin/S-1 Versus Cisplatin/5-FU: Secondary End Point Results From the First-Line Advanced Gastric Cancer Study (FLAGS). *Journal of gastrointestinal cancer [Online]* **2015,** *46* (2), 109–117.

Bowrey, D. J.; Baker, M.; Halliday, V.; Thomas, A. L.; Pulikottil-Jacob, R.; Smith, K.; Morris, T.; Ring, A. A randomised controlled trial of six weeks of home enteral nutrition versus standard care after oesophagectomy or total gastrectomy for cancer: report on a pilot and feasibility study. *Trials [Online]* **2015,** *16*, 531.

Castro Junior, G. de; Segalla, J. G.; Azevedo, S. J. de; Andrade, C. J.; Grabarz, D.; Araújo Lima França, B. de; Del Giglio, A.; Lazaretti, N. S.; Álvares, M. N.; Pedrini, J. L.; Kussumoto, C.; Matos Neto, J. N. de; Forones, N. M.; Fernandes Júnior, H. J.; Borges, G.; Girotto, G.; Da Silva, I. D. C. G.; Maluf-Filho, F.; Skare, N. G. A randomised phase II study of chemoradiotherapy with or without nimotuzumab in locally advanced oesophageal cancer: NICE trial. *European journal of cancer (Oxford, England: 1990) [Online]* **2018,** *88*, 21–30.

Dunne, D. F. J.; Jack, S.; Jones, R. P.; Jones, L.; Lythgoe, D. T.; Malik, H. Z.; Poston, G. J.; Palmer, D. H.; Fenwick, S. W. Randomized clinical trial of prehabilitation before planned liver resection. *The British journal of surgery [Online]* **2016,** *103* (5), 504–512.

Fretland, Å. A.; Dagenborg, V. J.; Bjørnelv, G. M. W.; Kazaryan, A. M.; Kristiansen, R.; Fagerland, M. W.; Hausken, J.; Tønnessen, T. I.; Abildgaard, A.; Barkhatov, L.; Yaqub, S.; Røsok, B. I.; Bjørnbeth, B. A.; Andersen, M. H.; Flatmark, K.; Aas, E.; Edwin, B. Laparoscopic Versus Open Resection for Colorectal Liver Metastases: The OSLO-COMET Randomized Controlled Trial. *Annals of surgery [Online]* **2018,** *267* (2), 199–207.

Froghi, F.; Sanders, G.; Berrisford, R.; Wheatley, T.; Peyser, P.; Rahamim, J.; Lewis, S. A randomised trial of post-discharge enteral feeding following surgical resection of an upper gastrointestinal malignancy. *Clinical nutrition (Edinburgh, Scotland) [Online]* **2017,** *36* (6), 1516–1519.

Gubanski, M.; Glimelius, B.; Lind, P. A. Quality of life in patients with advanced gastric cancer sequentially treated with docetaxel and irinotecan with 5-fluorouracil and folinic acid (leucovin). *Medical oncology (Northwood, London, England) [Online]* **2014,** *31* (4), 906.

Guo, Q.; Li, Q.; Wang, J.; Liu, M.; Wang, Y.; Chen, Z.; Ye, Y.; Guan, Q.; Zhou, Y. A comprehensive evaluation of clinical efficacy and safety of celecoxib in combination with chemotherapy in metastatic or postoperative recurrent gastric cancer patients: A preliminary, three-center, clinical trial study. *Medicine [Online]* **2019,** *98* (27), e16234.

Guo, W.-C.; Wang, F. Effect of nerve electrical stimulation for treating chemotherapy-induced nausea and vomiting in patients with advanced gastric cancer: A randomized controlled trial. *Medicine [Online]* **2018,** *97* (51), e13620.

Hafizi, M.; Kalanaky, S.; Moaiery, H.; Khayamzadeh, M.; Noorian, S.; Kaveh, V.; Gharib, B.; Foudazi, H.; Razavi, M.; Jenabian, A.; Salimi, S.; Sereshki, M. M. A.; Mirzaei, H. R.; Zarghi, A.; Fakharzadeh, S.; Nazaran, M. H.; Akbari, M. E. A randomized, double-blind, placebo-controlled investigation of BCc1 nanomedicine effect on survival and quality of life in metastatic and non-metastatic gastric cancer patients. *Journal of nanobiotechnology [Online]* **2019,** *17* (1), 52.

He, F.; Lin, X.; Xie, F.; Huang, Y.; Yuan, R. The effect of enhanced recovery program for patients undergoing partial laparoscopic hepatectomy of liver cancer. *Clinical & translational oncology: official publication of the Federation of Spanish Oncology Societies and of the National Cancer Institute of Mexico [Online]* **2015,** *17* (9), 694–701.

Ito, Y.; Yoshikawa, T.; Fujiwara, M.; Kojima, H.; Matsui, T.; Mochizuki, Y.; Cho, H.; Aoyama, T.; Ito, S.; Misawa, K.; Nakayama, H.; Morioka, Y.; Ishiyama, A.; Tanaka, C.; Morita, S.; Sakamoto, J.; Kodera, Y. Quality of life and nutritional consequences after aboral pouch reconstruction following total gastrectomy for gastric cancer: randomized controlled trial CCG1101. *Gastric cancer: official journal of the International Gastric Cancer Association and the Japanese Gastric Cancer Association [Online]* **2016,** *19* (3), 977–985.

Jin, Y.; Yong, C.; Ren, K.; Li, D.; Yuan, H. Effects of Post-Surgical Parenteral Nutrition on Patients with Gastric Cancer. *Cellular physiology and biochemistry: international journal of experimental cellular physiology, biochemistry, and pharmacology [Online]* **2018,** *49* (4), 1320–1328.

Kim, S. M.; Cho, J.; Kang, D.; Oh, S. J.; Kim, A. R.; Sohn, T. S.; Noh, J. H.; Kim, S. A Randomized Controlled Trial of Vagus Nerve-preserving Distal Gastrectomy Versus Conventional Distal Gastrectomy for Postoperative Quality of Life in Early Stage Gastric Cancer Patients. *Annals of surgery [Online]* **2016,** *263* (6), 1079–1084.

Koeberle, D.; Dufour, J.-F.; Demeter, G.; Li, Q.; Ribi, K.; Samaras, P.; Saletti, P.; Roth, A. D.; Horber, D.; Buehlmann, M.; Wagner, A. D.; Montemurro, M.; Lakatos, G.; Feilchenfeldt, J.; Peck-Radosavljevic, M.; Rauch, D.; Tschanz, B.; Bodoky, G. Sorafenib with or without everolimus in patients with advanced hepatocellular carcinoma (HCC): a randomized multicenter, multinational phase II trial (SAKK 77/08 and SASL 29). *Annals of oncology: official journal of the European Society for Medical Oncology [Online]* **2016,** *27* (5), 856–861.

Kong, S.-H.; Lee, H.-J.; Na, J.-R.; Kim, W. G.; Han, D.-S.; Park, S.-H.; Hong, H.; Choi, Y.; Ahn, H. S.; Suh, Y.-S.; Yang, H.-K. Effect of perioperative oral nutritional supplementation in malnourished patients who undergo gastrectomy: A prospective randomized trial. *Surgery [Online]* **2018,** *164* (6), 1263–1270.

Kouchaki, B.; Janbabai, G.; Alipour, A.; Ala, S.; Borhani, S.; Salehifar, E. Randomized double-blind clinical trial of combined treatment with megestrol acetate plus celecoxib versus megestrol acetate alone in cachexia-anorexia syndrome induced by GI cancers. *Supportive care in cancer: official journal of the Multinational Association of Supportive Care in Cancer [Online]* **2018,** *26* (7), 2479–2489.

Kripp, M.; Al-Batran, S.-E.; Rosowski, J.; Pauligk, C.; Homann, N.; Hartmann, J. T.; Moehler, M.; Hofheinz, R.-D. Quality of life of older adult patients receiving docetaxel-based chemotherapy triplets for esophagogastric adenocarcinoma: a randomized study

of the Arbeitsgemeinschaft Internistische Onkologie (AIO). *Gastric cancer: official journal of the International Gastric Cancer Association and the Japanese Gastric Cancer Association [Online]* **2014,** *17* (1), 181–187.

Kudo, M.; Finn, R. S.; Qin, S.; Han, K.-H.; Ikeda, K.; Piscaglia, F.; Baron, A.; Park, J.-W.; Han, G.; Jassem, J.; Blanc, J. F.; Vogel, A.; Komov, D.; Evans, T. R. J.; Lopez, C.; Dutcus, C.; Guo, M.; Saito, K.; Kraljevic, S.; Tamai, T.; Ren, M.; Cheng, A.-L. Lenvatinib versus sorafenib in first-line treatment of patients with unresectable hepatocellular carcinoma: a randomised phase 3 non-inferiority trial. *Lancet (London, England) [Online]* **2018,** *391* (10126), 1163–1173.

Lee, S. J.; Kim, S.; Kim, M.; Lee, J.; Park, Y. H.; Im, Y.-H.; Park, S. H. Capecitabine in combination with either cisplatin or weekly paclitaxel as a first-line treatment for metastatic esophageal squamous cell carcinoma: a randomized phase II study. *BMC cancer [Online]* **2015,** *15*, 693.

Li, L.; Mo, F.; Hui, E. P.; Chan, S. L.; Koh, J.; Tang, N. L. S.; Yu, S. C. H.; Yeo, W. The association of liver function and quality of life of patients with liver cancer. *BMC gastroenterology [Online]* **2019,** *19* (1), 66.

Lu, Z.; Zhang, X.; Liu, W.; Liu, T.; Hu, B.; Li, W.; Fan, Q.; Xu, J.; Xu, N.; Bai, Y.; Pan, Y.; Xu, Q.; Bai, W.; Xia, L.; Gao, Y.; Wang, W.; Shu, Y.; Shen, L. A multicenter, randomized trial comparing efficacy and safety of paclitaxel/capecitabine and cisplatin/capecitabine in advanced gastric cancer. *Gastric cancer: official journal of the International Gastric Cancer Association and the Japanese Gastric Cancer Association [Online]* **2018,** *21* (5), 782–791.

Lv, N.; Kong, Y.; Mu, L.; Pan, T.; Xie, Q.; Zhao, M. Effect of perioperative parecoxib sodium on postoperative pain control for transcatheter arterial chemoembolization for inoperable hepatocellular carcinoma: a prospective randomized trial. *European radiology [Online]* **2016,** *26* (10), 3492–3499.

Miyakawa, A.; Kodera, S.; Sakuma, Y.; Shimada, T.; Kubota, M.; Nakamura, A.; Itobayashi, E.; Shimura, H.; Suzuki, Y.; Sato, Y.; Shimura, K. Effects of Early Initiation of Solid Versus Liquid Diet after Endoscopic Submucosal Dissection on Quality of Life and Postoperative Outcomes: A Prospective Pilot Randomized Controlled Trial. *Digestion [Online]* **2019,** *100* (3), 160–169.

Mudge, L. A.; Watson, D. I.; Smithers, B. M.; Isenring, E. A.; Smith, L.; Jamieson, G. G. Multicentre factorial randomized clinical trial of perioperative immunonutrition versus standard nutrition for patients undergoing surgical resection of oesophageal cancer. *The British journal of surgery [Online]* **2018,** *105* (10), 1262–1272.

Nakamura, M.; Nakamori, M.; Ojima, T.; Iwahashi, M.; Horiuchi, T.; Kobayashi, Y.; Yamade, N.; Shimada, K.; Oka, M.; Yamaue, H. Randomized clinical trial comparing long-term quality of life for Billroth I versus Roux-en-Y reconstruction after distal gastrectomy for gastric cancer. *The British journal of surgery [Online]* **2016,** *103* (4), 337–347.

O'Neill, L. M.; Guinan, E.; Doyle, S. L.; Bennett, A. E.; Murphy, C.; Elliott, J. A.; O'Sullivan, J.; Reynolds, J. V.; Hussey, J. The RESTORE Randomized Controlled Trial: Impact of a Multidisciplinary Rehabilitative Program on Cardiorespiratory Fitness in Esophagogastric cancer Survivorship. *Annals of surgery [Online]* **2018,** *268* (5), 747–755.

Pereira, H.; Bouattour, M.; Dioguardi Burgio, M.; Assenat, E.; Grégory, J.; Bronowicki, J.-P.; Chatellier, G.; Vilgrain, V. Health-related quality of life in locally advanced hepatocellular carcinoma treated by either radioembolisation or sorafenib (SARAH trial). *European journal of cancer (Oxford, England: 1990) [Online]* **2021,** *154*, 46–56.

Román, E.; Torrades, M. T.; Nadal, M. J.; Cárdenas, G.; Nieto, J. C.; Vidal, S.; Bascuñana, H.; Juárez, C.; Guarner, C.; Córdoba, J.; Soriano, G. Randomized pilot study: effects of an exercise programme and leucine supplementation in patients with cirrhosis. *Digestive diseases and sciences [Online]* **2014,** *59* (8), 1966–1975.

Ryoo, B.-Y.; Merle, P.; Kulkarni, A. S.; Cheng, A.-L.; Bouattour, M.; Lim, H. Y.; Breder, V.; Edeline, J.; Chao, Y.; Ogasawara, S.; Yau, T.; Garrido, M.; Chan, S. L.; Daniele, B.; Norquist, J. M.; Chen, E.; Siegel, A. B.; Zhu, A. X.; Finn, R. S.; Kudo, M. Health-related quality-of-life impact of pembrolizumab versus best supportive care in previously systemically treated patients with advanced hepatocellular carcinoma: KEYNOTE-240. *Cancer [Online]* **2021,** *127* (6), 865–874.

Scarpi, E.; Dall'Agata, M.; Zagonel, V.; Gamucci, T.; Bertè, R.; Sansoni, E.; Amaducci, E.; Broglia, C. M.; Alquati, S.; Garetto, F.; Schiavon, S.; Quadrini, S.; Orlandi, E.; Casadei Gardini, A.; Ruscelli, S.; Ferrari, D.; Pino, M. S.; Bortolussi, R.; Negri, F.; Stragliotto, S.; Narducci, F.; Valgiusti, M.; Farolfi, A.; Nanni, O.; Rossi, R.; Maltoni, M. Systematic vs. on-demand early palliative care in gastric cancer patients: a randomized clinical trial assessing patient and healthcare service outcomes. *Supportive care in cancer: official journal of the Multinational Association of Supportive Care in Cancer [Online]* **2019,** *27* (7), 2425–2434.

Snoeren, N.; van Hillegersberg, R.; Schouten, S. B.; Bergman, A. M.; van Werkhoven, E.; Dalesio, O.; Tollenaar, R. A. E. M.; Verheul, H. M.; van der Sijp, J.; Borel Rinkes, I. H. M.; Voest, E. E. Randomized Phase III Study to Assess Efficacy and Safety of Adjuvant CAPOX with or without Bevacizumab in Patients after Resection of Colorectal Liver Metastases: HEPATICA study. *Neoplasia (New York, N.Y.) [Online]* **2017,** *19* (2), 93–99.

So, J. B.-Y.; Rao, J.; Wong, A. S.-Y.; Chan, Y.-H.; Pang, N. Q.; Tay, A. Y. L.; Yung, M. Y.; Su, Z.; Phua, J. N. S.; Shabbir, A.; Ng, E. K. W. Roux-en-Y or Billroth II Reconstruction After Radical Distal Gastrectomy for Gastric Cancer: A Multicenter Randomized Controlled Trial. *Annals of surgery [Online]* **2018,** *267* (2), 236–242.

Sun, D.; Jiao, J.; Zhang, X.; Xu, J.; Ye, M.; Xiu, L.; Zhao, Y.; Lu, Y.; Liu, X.; Zhao, J.; Shi, J.; Qin, Z.; Wei, P. Therapeutic effect of Jinlongshe Granule on quality of life of stage IV gastric cancer patients using EORTC QLQ-C30: A double-blind placebo-controlled clinical trial. *Chinese journal of integrative medicine [Online]* **2015,** *21* (8), 579–586.

Uitdehaag, M. J.; van Putten, P. G.; van Eijck, C. H. J.; Verschuur, E. M. L.; van der Gaast, A.; Pek, C. J.; van der Rijt, C. C. D.; Man, R. A. de; Steyerberg, E. W.; Laheij, R. J. F.; Siersema, P. D.; Spaander, M. C. W.; Kuipers, E. J. Nurse-led follow-up at home vs. conventional medical outpatient clinic follow-up in patients with incurable upper gastrointestinal cancer: a randomized study. *Journal of pain and symptom management [Online]* **2014,** *47* (3), 518–530.

Wang, H.; Hu, X.; Chen, S.; Xiang, J.; Yang, Z.; Zhou, Z.; Chen, Y.; Lin, Y.; Chen, Y.; Peng, J. Functional jejunal interposition versus Roux-en-Y anastomosis after total gastrectomy for gastric cancer: A prospective randomized clinical trial. *Surgical oncology [Online]* **2020,** *34*, 236–244.

Wang, J.; Yan, C.; Fu, A. A randomized clinical trial of comprehensive education and care program compared to basic care for reducing anxiety and depression and improving quality of life and survival in patients with hepatocellular carcinoma who underwent surgery. *Medicine [Online]* **2019,** *98* (44), e17552.

Woo, J.; Lee, J.-H.; Shim, K.-N.; Jung, H.-K.; Lee, H. M.; Lee, H. K. Does the Difference of Invasiveness between Totally Laparoscopic Distal Gastrectomy and Laparoscopy-Assisted Distal Gastrectomy Lead to a Difference in Early Surgical Outcomes? A Prospective Randomized Trial. *Annals of surgical oncology [Online]* **2015,** *22* (6), 1836–1843.

Ye, X.; Lu, D.; Chen, X.; Li, S.; Chen, Y.; Deng, L. A Multicenter, Randomized, Double-Blind, Placebo-Controlled Trial of Shuangbai San for Treating Primary Liver Cancer Patients With Cancer Pain. *Journal of pain and symptom management [Online]* **2016,** *51* (6), 979–986.

Yoshino, S.; Nishikawa, K.; Morita, S.; Takahashi, T.; Sakata, K.; Nagao, J.; Nemoto, H.; Murakami, N.; Matsuda, T.; Hasegawa, H.; Shimizu, R.; Yoshikawa, T.; Osanai, H.; Imano, M.; Naitoh, H.; Tanaka, A.; Tajiri, T.; Gochi, A.; Suzuki, M.; Sakamoto, J.; Saji, S.; Oka, M. Randomised phase III study of S-1 alone versus S-1 plus lentinan for unresectable or recurrent gastric cancer (JFMC36-0701). *European journal of cancer (Oxford, England: 1990) [Online]* **2016,** *65*, 164–171.

Zaman, A. C. G. N. M.; Tytgat, K. M. A. J.; Klinkenbijl, J. H. G.; Boer, F. C. den; Brink, M. A.; Brinkhuis, J. C.; Bruinvels, D. J.; Dol, L. C. M.; van Duijvendijk, P.; Hemmer, P. H. J.; Lamme, B.; Loosveld, O. J. L.; Mok, M. M.; Rejda, T.; Rutten, H.; Schoorlemmer, A.; Sonneveld, D. J.; Stassen, L. P. S.; Veenstra, R. P.; van de Ven, A.; Velzing, E. R.; Frings-Dresen, M. H. W.; Boer, A. G. E. M. de. Effectiveness of a Tailored Work-Related Support Intervention for Patients Diagnosed with Gastrointestinal Cancer: A Multicenter Randomized Controlled Trial. *Journal of occupational rehabilitation [Online]* **2021,** *31* (2), 323–338.

Zhu, A. X.; Macarulla, T.; Javle, M. M.; Kelley, R. K.; Lubner, S. J.; Adeva, J.; Cleary, J. M.; Catenacci, D. V. T.; Borad, M. J.; Bridgewater, J. A.; Harris, W. P.; Murphy, A. G.; Oh, D.-Y.; Whisenant, J. R.; Lowery, M. A.; Goyal, L.; Shroff, R. T.; El-Khoueiry, A. B.; Chamberlain, C. X.; Aguado-Fraile, E.; Choe, S.; Wu, B.; Liu, H.; Gliser, C.; Pandya, S. S.; Valle, J. W.; Abou-Alfa, G. K. Final Overall Survival Efficacy Results of Ivosidenib for Patients With Advanced Cholangiocarcinoma With IDH1 Mutation: The Phase 3 Randomized Clinical ClarIDHy Trial. *JAMA oncology [Online]* **2021,** *7* (11), 1669–1677.

Zhu, M.-W.; Yang, X.; Xiu, D.-R.; Yang, Y.; Li, G.-X.; Hu, W.-G.; Wang, Z.-G.; Cui, H.-Y.; Wei, J.-M. Effect of oral nutritional supplementation on the post-discharge nutritional status and quality of life of gastrointestinal cancer patients after surgery: a multi-center study. *Asia Pacific journal of clinical nutrition [Online]* **2019,** *28* (3), 450–456.
